# Supplementary material for: The impact of physiological state and environmental stress on bacterial load estimation methodologies for Mycobacterium tuberculosis
Source: Sci Rep. 2024 Oct 30;14:26108. doi: 10.1038/s41598-024-74318-3 (PMC11525806; doi:10.1038/s41598-024-74318-3)
Supplement: Supplementary file 6 — Supplementary Material 6 [file 41598_2024_74318_MOESM6_ESM.pdf]

Title: The impact of physiological state and environmental stress on bacterial load estimation methodologies for *Mycobacterium tuberculosis*

Authors: Arundhati Maitra[1,2‡], Marie Wijk[1,3‡*], Hasmik Margaryan[2], Paolo Denti[3], Timothy D. McHugh[2], Frank Kloprogge[1,2]

Author affiliations: [1] Institute for Global Health, University College London, London, United Kingdom [2] Centre for Clinical Microbiology, University College London, London, United Kingdom [3] Division of Clinical Pharmacology, Department of Medicine, University of Cape Town, Cape Town, South Africa

[‡]These authors contributed equally.

Corresponding author details: Marie Wijk. Division of Clinical Pharmacology, Department of Medicine, University of Cape Town, Cape Town, South Africa.

Email: sjlmar001@myuct.ac.za

Phone: +27663343175.

Description of the models used in the study

A. Analysis of relationship between TTP and CFU

A linear model estimating an intercept and slope adequately described the relationship between TTP and CFU (Fig. 1). Typical values for intercept and slope were estimated to 215 hours and -50.3 hours/log10 CFU, respectively (Table 1). Between-biological replicate variability was included on the intercept and an additive error model on logarithmic scale described the residual variability. Additive error was inflated by 1.94-fold for samples with <10 colonies (dOFV = -22.8, degrees of freedom, df = 1, $p < 0.001$). Accounting for the additional error introduced when reading plates with >100 CFU did not further improve the fit (dOFV = -1.81, df = 1, p = 0.18) and was thus not kept in the model. The presence of antibiotic significantly affected the intercept, with antibiotic exposure resulting in 17.6% lower intercept (dOFV = -7.33, df = 1, p = 0.007). Samples cultured on day 11 or 21 had 15.3% lower intercept (dOFV = -11.3, df = 1, $p < 0.001$) compared to samples cultured on day 0 or 3. A VPC stratified by antibiotic presence and culture-age can be found in Fig. 1. Similar results were found in the dataset with imputed CFUs (Supplementary Fig. S1, Supplementary Table S1), with the difference of samples with culture-age of 0, 11 or 21 days had 18.6-folds larger random variability than samples with culture-age of 3 days (difference in OFV, dOFV = -7.92) in the data with no imputations.

B. Analysis of relationship between TTG and CFU

The relationship between TTG and CFU was adequately described by a linear model estimating an intercept and slope to 184 hours and -42.2 hours/log10 CFU, respectively (Fig. 2 and Table 2). Between-biological replicate variability was included on the intercept and an additive error model on logarithmic scale was used to describe the residual variability. Culture-age significantly affected TTG for the same CFU with 18.3% lower intercept for culture ages of 11 and 21 days than that for those 0 and 3 days (dOFV = -25.7, df = 1, $p < 0.001$). The presence of antibiotics did not have any impact on the relationship between TTG and CFU. A VPC stratified by culture-age can be found in Fig. 2. Similar results were found in the dataset with imputed CFUs (Supplementary Fig. S2, Supplementary Table S2).

C. Analysis of relationship between GU and incubation time in MGIT

A total of 184,129 observations from 227 experiments were included in the analysis (Fig. 3). A logistic model estimating an asymptote, a slope, and the time of the function's midpoint (T50) fit the data best. Typical values for the asymptote, slope and T50 were 12,800 GU, 0.069 GU/hour and 88.9 hours, respectively (Table 3). Random variability was included on all three parameters and an additive error model on logarithmic scale described the residual variability. Estimating covariance between the random variabilities in the slope and T50 further improved the model fit (dOFV = -445). Culture-age significantly affected the slope and asymptote, with samples cultured on day 11 or 21 having 6.18% bigger slope (dOFV = -8.15, df = 1, p = 0.004) and 23.6% higher asymptote (dOFV = -8.78, df = 1, p = 0.003) compared to samples cultured on day 0 or 3. A VPC stratified by culture-age is shown in Fig. 3.

Figures and Tables

Supplementary Table S1: Parameter estimates for model describing relationship between TTP and CFU in dataset with imputations.

Parameter	Typical value (95% CI)[a]
Intercept (hours)	228 (209 – 250)
Slope (hours/log10 CFU)	-28.7 (-31.3 – -26.2)
Additive error on log scale (%)	13.9 (12.5 – 15.7)
Scaling of additive error on log scale for < 10 CFU (folds)	1.90 (1.54 – 2.43)
Between-biological replicate variability on intercept (%CV)	9.54 (6.52 – 12.4)
Effect of antibiotic presence on intercept (%)	-21.7 (-34.5 – -9.02)
Effect of culture-age 11 or 21 days on intercept (%)	-10.4 (-13.9 – -6.87)

[a] 95% confidence interval obtained by sampling importance resampling (SIR) using Pearl-Speaks-NONMEM.

Supplementary Table S2: Parameter estimates for model describing relationship between TTG and CFU in dataset with imputations.

**Parameter**	**Typical value (95% CI)[a]**
Intercept (hours)	186 (172 – 200)
Slope (hours/log10 CFU)	-30.9 (-34.3 – -27.2)
Additive error on log scale (%)	20.2 (18.3 – 22.8)
Between-biological replicate variability on intercept (%CV)	7.07 (4.34 – 9.50)
Effect of culture-age 11 and 21 days on the intercept (%)	-13.0 (-18.3 – -7.68)

[a] 95% confidence interval obtained by sampling importance resampling (SIR) using Pearl-Speaks-NONMEM.

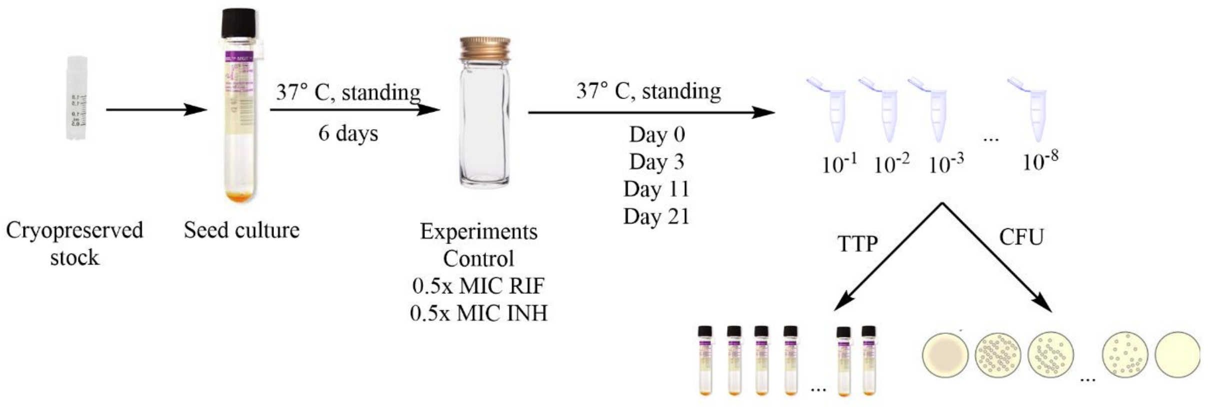

Supplementary Figure S1. Schematic representation of the experimental design.

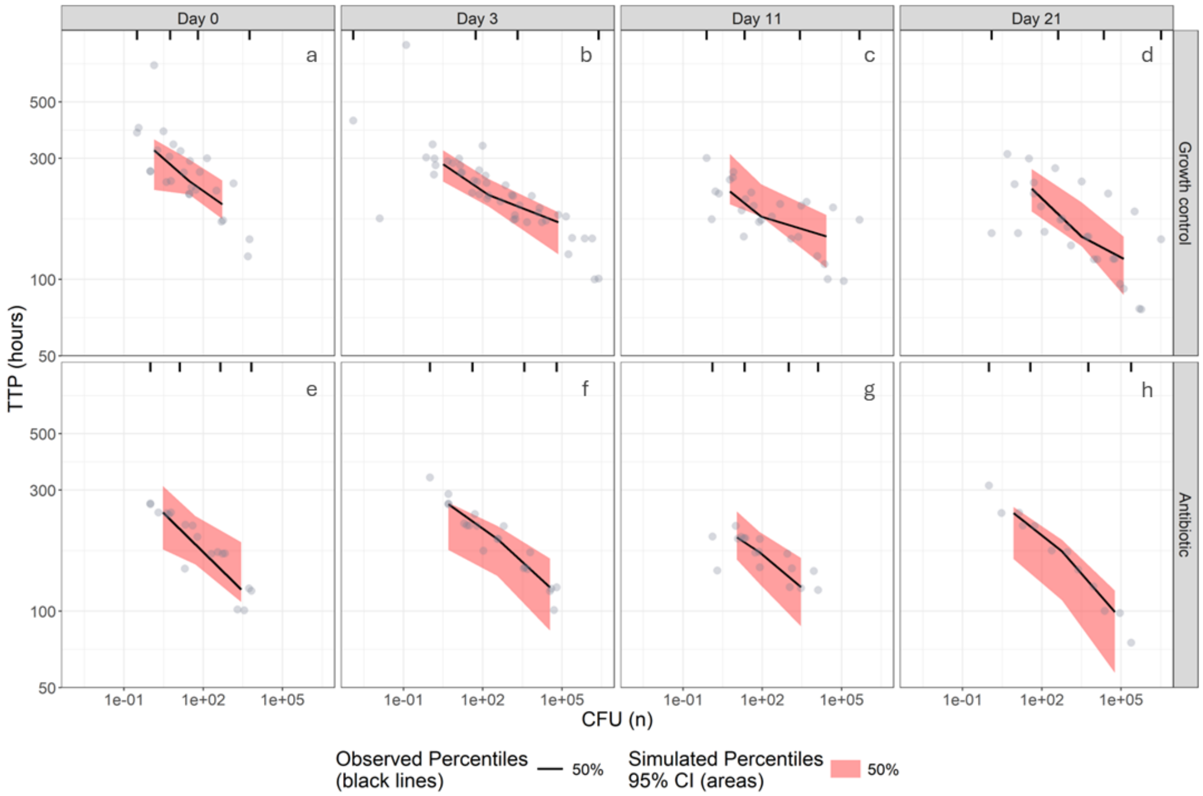

Supplementary Figure S2. Visual predictive check (n=1000) of TTP versus CFU in dataset with imputations, stratified by culture-age and presence of antibiotic. The circles represent observations. The rows represent absence (panels a-d) or presence (panels e-f) of antibiotic, whereas the columns represent the age of the cultures.

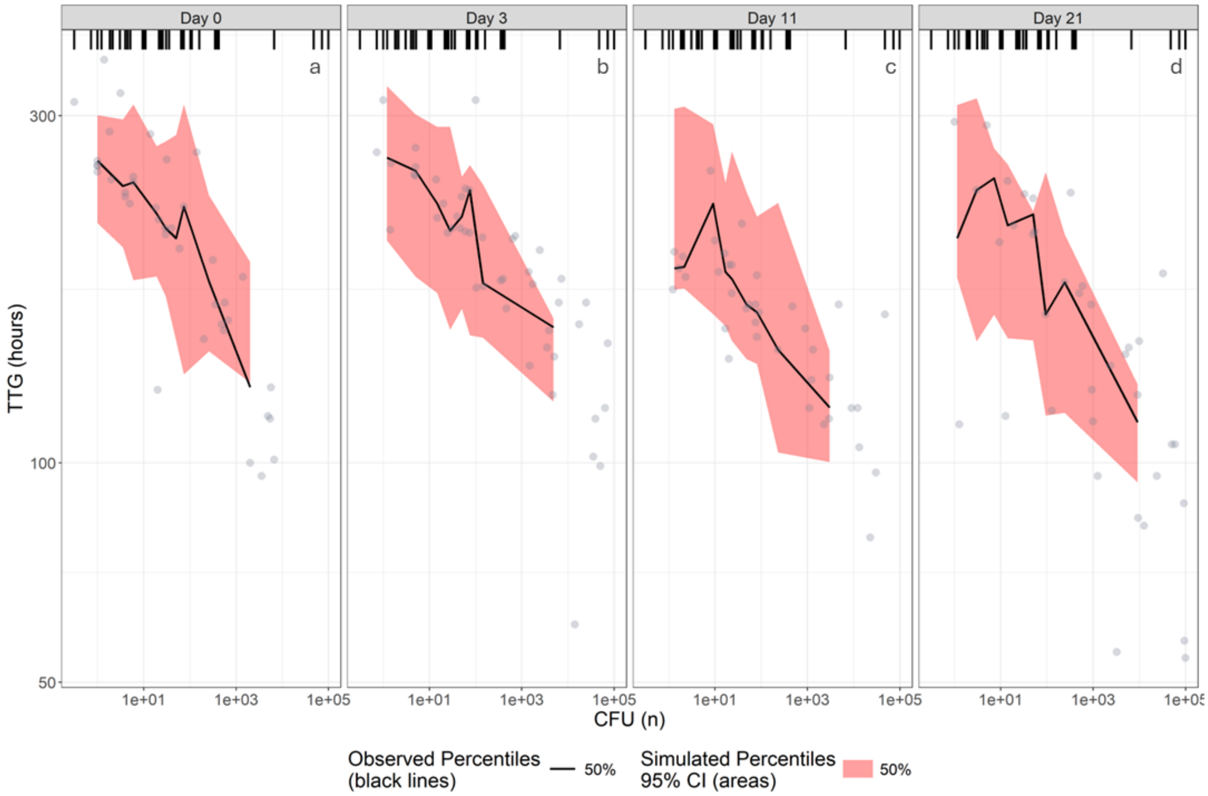

Supplementary Figure S3. Visual predictive check (n=1000) of TTG versus CFU in dataset with imputations, stratified by culture-age. The circles represent observations, and the columns represent the age of the cultures.

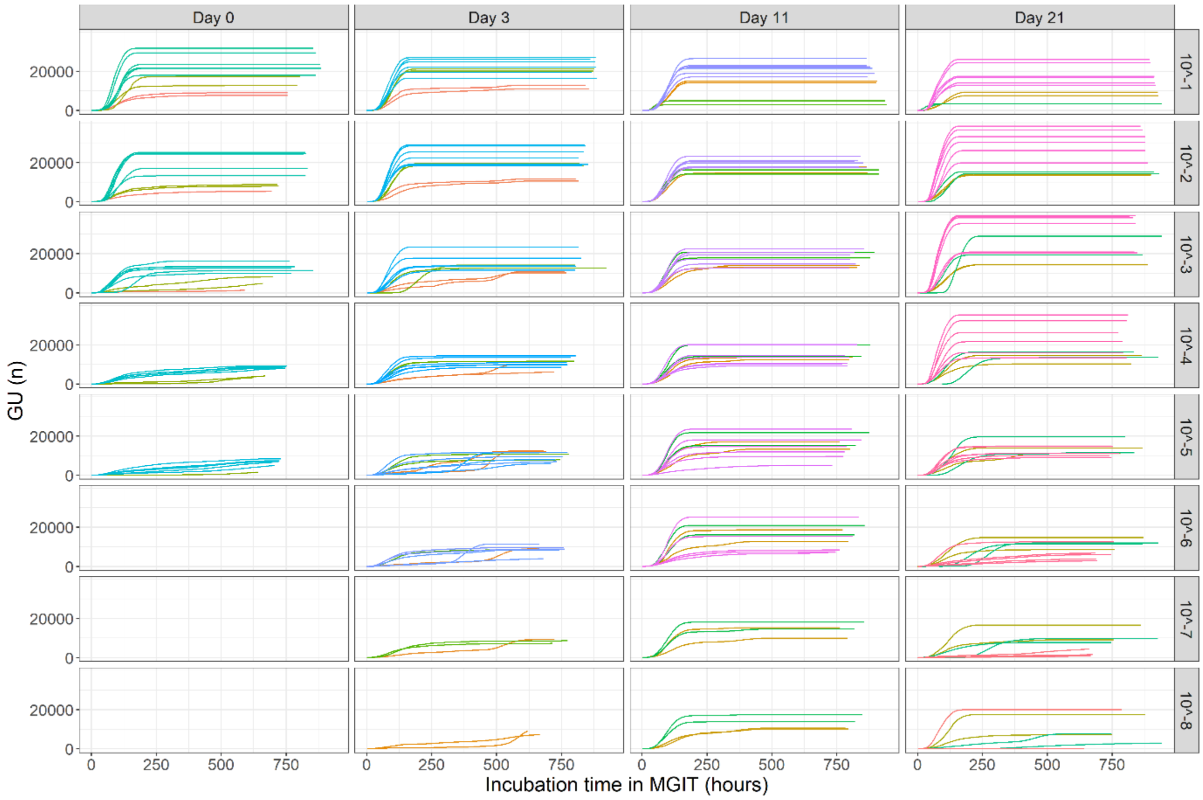

Supplementary Figure S4. Raw GU data stratified by culture-age and dilution. Each trajectory represents GU reads from one MGIT.

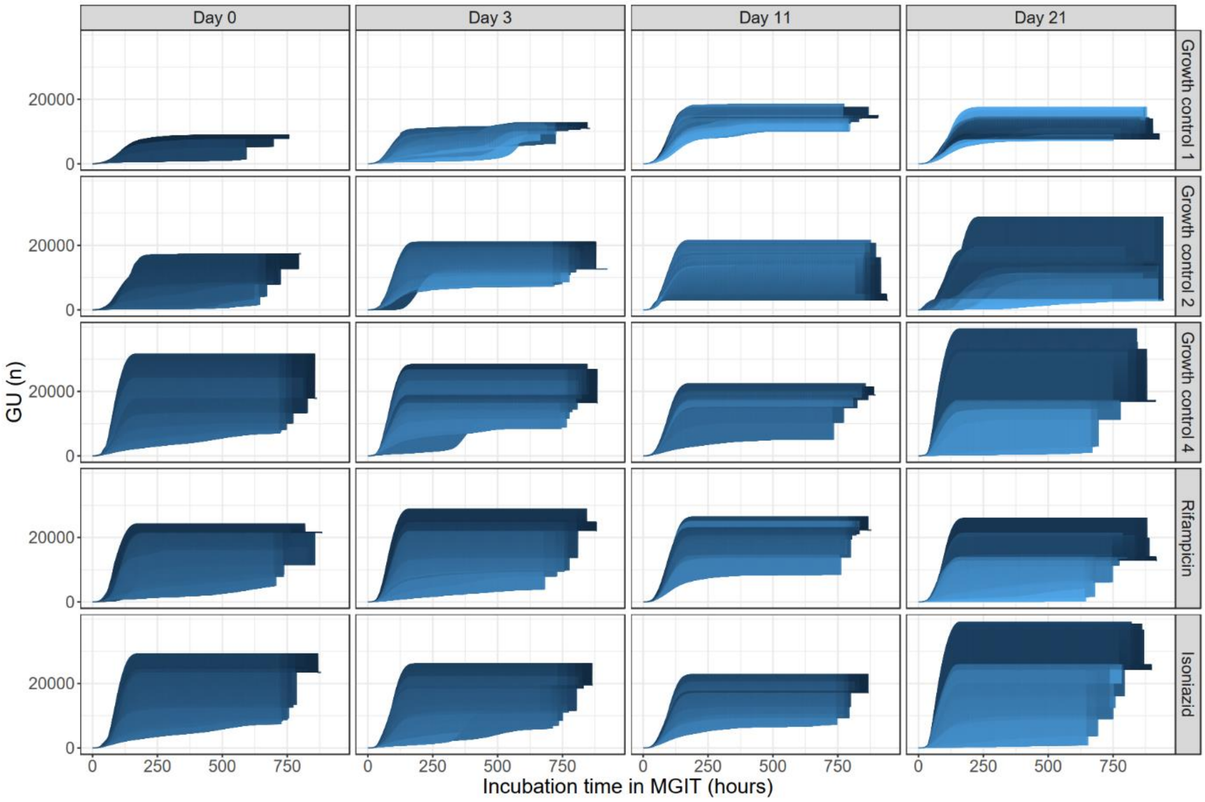

Supplementary Figure S5. Raw GU data stratified by culture-age and biological replicate and coloured by dilution, where the lighter lines represent more diluted samples. Each trajectory represents GU reads from one MGIT.
